# Assessing the performance of trūRapid™ FOUR heartworm antigen test for early detection in experimentally infected dogs in comparison to other point-of-care tests

**DOI:** 10.1186/s13071-026-07450-w

**Published:** 2026-08-20

**Authors:** Pabasara Weerarathne, Tiana L. Sanders, Alexa Starnes, Maureen A. Kelly, Pablo D. Jimenez Castro, Frances M. Moore, Demitria M. Vasilatis, Christian M. Leutenegger, Chinta Lamichhane, Haichen Song, Guilherme G. Verocai

**Affiliations:** 1 Department of Veterinary Pathobiology, College of Veterinary Medicine and Biomedical Sciences, Texas A&M University, College Station, TX USA; 2 Antech Diagnostics, Mars Petcare Science & Diagnostics, Loveland, CO USA

**Keywords:** Heartworm disease, Point-of-care, trūrapid™ FOUR, Antigen detection test, Dogs

## Abstract

**Background:**

The American Heartworm Society (AHS) recommends diagnosing canine heartworm (HW) infection using both antigen and microfilaria detection tests. Antigen–antibody immune complexes may block antigen detection. Therefore, the AHS recommends immune complex dissociation (ICD) via heat treatment for discordant cases. The trūRapid™ FOUR antigen test kit (Antech Diagnostics) is a novel commercially available point-of-care (POC) device. This study evaluated the performance of trūRapid™ FOUR compared to other commercially available POC tests, namely SNAP^®^ 4Dx^®^ Plus (IDEXX), VETSCAN^®^ Flex4 (Zoetis), and a plate-based assay, DiroCHEK^®^ (Zoetis), for early detection of HW infection in experimentally infected dogs.

**Methods:**

Archival frozen serum samples from six purpose-bred beagles experimentally infected with *Dirofilaria immitis* were collected weekly for 5.5 months, from week 1 to week 22 post-infection. Sera were tested for the detection of HW antigen, pre- and post-ICD, via heat treatment (103 °C for 10 min in a heat block) using each of the commercial antigen tests. DiroCHEK^®^ results were interpreted using optical density readings.

**Results:**

HW antigen was not detected in samples collected from week 1 through week 19. In week 20, the trūRapid™ FOUR, SNAP^®^ 4Dx^®^ Plus, and VETSCAN^®^ Flex4 detected HW antigen in one out of six samples (16.7%) post-ICD, while DiroCHEK^®^ detected two (33.3%) post-ICD. In week 21, the trūRapid™ FOUR, SNAP^®^ 4Dx^®^ Plus, and DiroCHEK^®^ detected HW antigen in four out of the five (80%) post-ICD samples, whereas the VETSCAN^®^ Flex4 detected three out of the four (75%) post-ICD samples. In week 22, HW antigen was detected in one of six (16.7%) pre-ICD by all tests; trūRapid™ FOUR, SNAP^®^ 4Dx^®^ Plus, and DiroCHEK^®^ detected HW antigen on all six (100%) samples post-ICD, while VETSCAN^®^ Flex4 detected four of six samples (66.7%).

**Conclusions:**

Our findings indicate that trūRapid™ FOUR and other commercially available antigen detection tests can detect HW antigen as early as 5 months post-infection when serum is subjected to heat treatment. The performance of trūRapid™ FOUR is comparable to other widely used commercial antigen kits. Its ease of use and ability to detect HW infection earlier in the course of antigenemia makes it an excellent POC diagnostic tool, allowing for prompt clinical intervention, and increasing patient quality of life.

**Graphical Abstract:**

**Figure:**
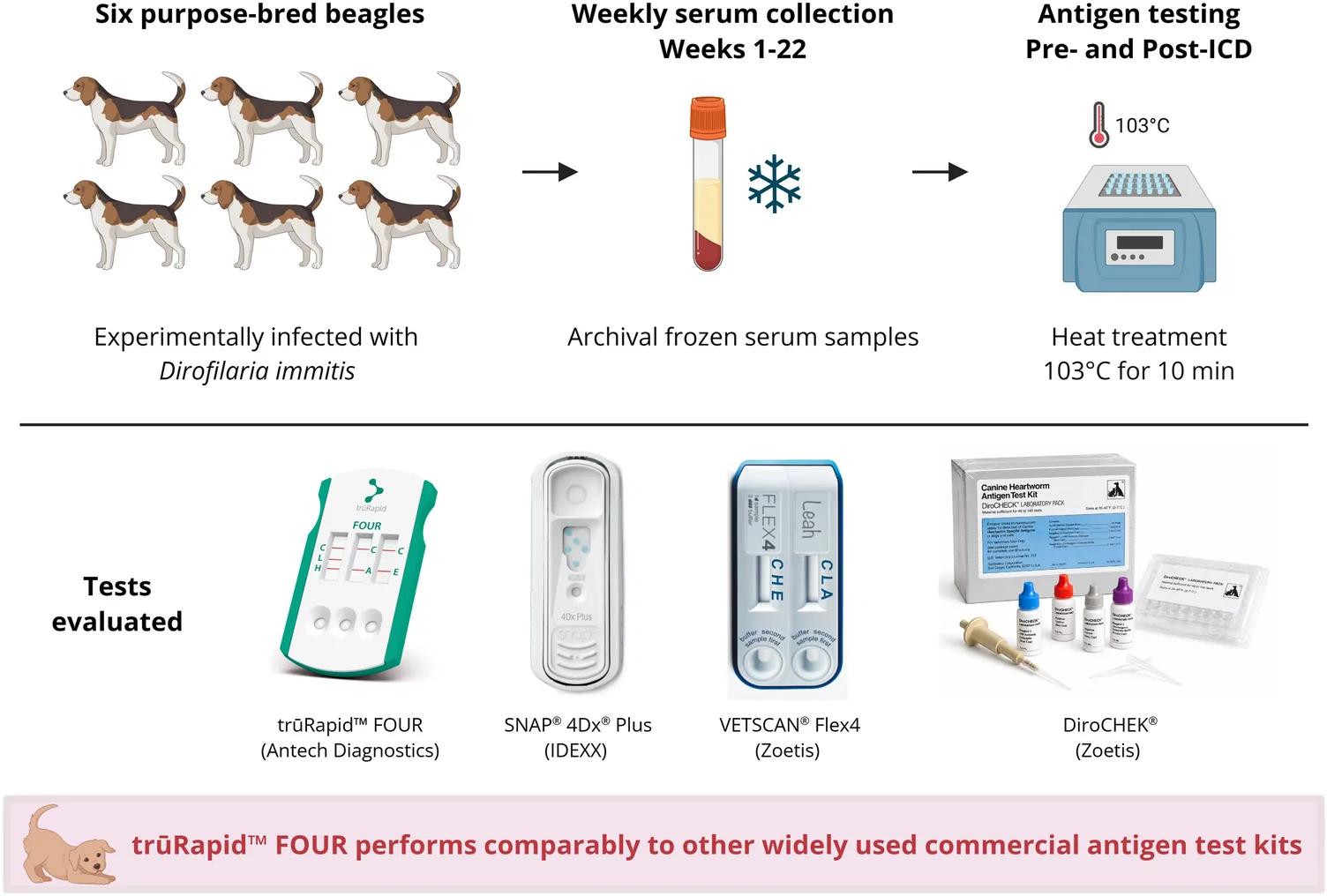

## Background

Canine heartworm (HW) disease, caused by the mosquito-borne filarial nematode *Dirofilaria immitis*, is a serious and potentially fatal disease affecting domestic dogs worldwide [1–5]. In the United States, based on current data from commercial laboratories reported to the Companion Animal Parasite Council (2025), one in every 100 dogs tests positive for *D. immitis* infection [6]. Following transmission of third-stage larvae (L3) by mosquito vectors, the developing worms migrate to the right ventricle and pulmonary arteries, maturing into young adults within about 4 months [1, 4]. By 6 months post-infection, female worms are typically fully developed and begin releasing microfilariae into the host circulatory system [4]. Adult HWs residing in pulmonary arteries cause vascular and pulmonary pathology characterized by villous proliferation, endarteritis, and fibrosis, leading to pulmonary hypertension and right-sided heart enlargement [4, 7, 8]. Severe infections may progress to right-sided heart failure or, in rare cases, to caval syndrome, a life-threatening condition resulting from mechanical obstruction of the right atrium and tricuspid valve by adult worms, causing hemolytic anemia and circulatory collapse [7, 9]. Additional complications include thromboembolic events following worm death, glomerulonephritis from immune complex deposition, and less commonly, cutaneous lesions associated with circulating microfilariae [7, 10].

The clinical course of HW infection is often insidious, and early infections can remain undetected for months [1, 7]. It typically takes 5 to 7 months post-infection before adult female worms begin producing circulating antigen detectable by available commercial diagnostic tests. Dogs are often asymptomatic during this prepatent period, yet microscopic vascular and inflammatory changes are already occurring [11, 12]. Early detection is therefore essential, as prompt initiation of adulticidal therapy can improve clinical outcomes and minimize long-term cardiopulmonary damage [1, 7, 13]. To address this, the American Heartworm Society (AHS) recommends annual screening of all dogs aged 7 months or older using both an antigen and a microfilaria detection test [11].

Currently, the mainstay of HW diagnosis in clinical settings is the use of antigen-based tests, which detect a glycoprotein secreted by adult female *D. immitis* [14–16]. These tests are commonly implemented as point-of-care (POC) immunochromatographic assays, including widely used platforms such as SNAP^®^ 4Dx^®^ Plus (IDEXX, Westbrook, ME, USA), VETSCAN^®^ Flex4 (Zoetis, Parsippany, NJ, USA), and others. While these assays are highly specific and generally sensitive in dogs with established infections, their diagnostic performance can be compromised in certain cases. These include infections with low worm burden (e.g., only one or two females), male-only infections, or cases in which circulating antigen is sequestered in immune complexes [14, 15, 17–19].

The formation of antigen–antibody complexes can interfere with detection by standard assays, leading to false-negative results despite active infection [20, 21]. To overcome this issue, immune complex dissociation (ICD) methods, most notably heat treatment, have been employed to release bound antigen, thereby enhancing test sensitivity [21–23]. Several studies have demonstrated that ICD can reveal previously undetectable antigen in both experimentally and naturally infected dogs [15, 24–29]. As a result, the AHS supports the use of ICD when results are inconsistent with clinical suspicion, particularly in high-risk patients or those with a history of preventive lapse [11].

Despite its utility, ICD is generally limited to reference laboratory settings due to practical and technical constraints. The process requires special equipment such as calibrated heat blocks and centrifuges. These requirements are typically not feasible in routine veterinary practice. Meanwhile, clinicians rely on POC tests for immediate, in-clinic decision-making. Newer POC platforms continue to emerge, aiming to improve diagnostic efficiency and sensitivity while maintaining ease of use. The trūRapid™ FOUR (Antech Diagnostics, Loveland, CO, USA) is a recently introduced lateral flow assay that detects *D. immitis* antigen along with antibodies to *Borrelia burgdorferi*, *Anaplasma* spp., and *Ehrlichia* spp., enabling multiplex vector-borne disease screening [30]. However, its performance for early HW detection has not yet been evaluated in experimentally infected dogs, nor has it been compared to other established POC tests in cases where antigen is sequestered within immune complexes.

The objective of this study was to evaluate the performance of the trūRapid™ FOUR antigen test in detecting HW infection during the early post-infection period in experimentally infected dogs, both with and without ICD. Results were compared with those of three other commercially available antigen tests, namely SNAP^®^ 4Dx^®^ Plus, VETSCAN^®^ Flex4, and the laboratory-based DiroCHEK^®^ enzyme-linked immunosorbent assay (ELISA; Zoetis, Parsippany, NJ, USA), to determine relative sensitivity and timing of antigen detection.

## Methods

### Samples

A set of archival serum samples collected weekly from a previous study, not conducted at Antech Diagnostics, were used in the present study. Six purpose-bred beagles were each inoculated subcutaneously with 50 infective L3 of *D. immitis* (Metairie isolate [31]). Whole blood samples were collected weekly from week 0 through week 22 into red-top tubes and allowed to clot for serum separation. Aliquots of serum were stored at −80 °C until used for testing.

### Serological testing

#### Heat treatment

Each serum sample was thawed, vortexed, and centrifuged at 1700×*g* for 5 min prior to testing. To dissociate immune complexes, 400 µl of each sample was transferred to a labeled 1.5-ml Eppendorf tube and heated at 103 °C for 10 min in a heat block. Following incubation, samples were centrifuged at 16,000×*g* for 10 min [32–34]. The resulting supernatants were designated as “post-ICD” and used to evaluate the effect of ICD on test results. The corresponding non-heat-treated samples were designated as “pre-ICD.”

#### Comparison of heartworm antigen tests

Each pre- and post-ICD sample (volume permitting) was tested in parallel, using trūRapid™ FOUR (Antech Diagnostics, Loveland, CO, USA), SNAP^®^ 4Dx^®^ Plus (IDEXX, Westbrook, ME, USA), VETSCAN^®^ Flex4 (Zoetis, Parsippany, NJ, USA), and DiroCHEK^®^ (Zoetis, Parsippany, NJ, USA) according to the manufacturer’s instructions. Each sample was tested once with each assay, and results were recorded as antigen-positive or no antigen detected (NAD). For DiroCHEK^®^, both visual interpretation and spectrophotometric optical density (OD) readings were obtained at 490 nm using a microplate reader (Synergy H1, BioTek Instruments, Inc., Winooski, VT, USA). During visual interpretation, the sample was considered positive if a clear to blue color change occurred 5 min following the addition of the final solution. Otherwise, the sample was considered as NAD for HW infection. For spectrophotometric readings, samples that had OD values greater than the concurrently tested negative control were considered positive for HW. Photographs of all test results (pre-ICD and post-ICD) were captured for visual comparison.

### Statistical analysis

Due to insufficient serum volume, results were unavailable for two dogs; therefore, paired analyses were conducted using only the dogs with complete datasets (*n* = 4/6). Cochran’s *Q* test was used to evaluate the differences between the proportion of antigen-positive results among the assays tested (trūRapid™ FOUR, SNAP^®^ 4Dx^®^ Plus, VETSCAN^®^ Flex4, and DiroCHEK^®^) across time points, both pre-ICD and post-ICD. A post hoc analysis was also performed using McNemar’s test with Bonferroni correction applied to evaluate each pair of tests. Unless otherwise defined, statistical significance was defined as *P* < 0.05. All analyses were performed using STATA^®^ version 19.5 Basic Edition (StataCorp LLC, College Station, TX, USA).

## Results

**Fig. 1:**
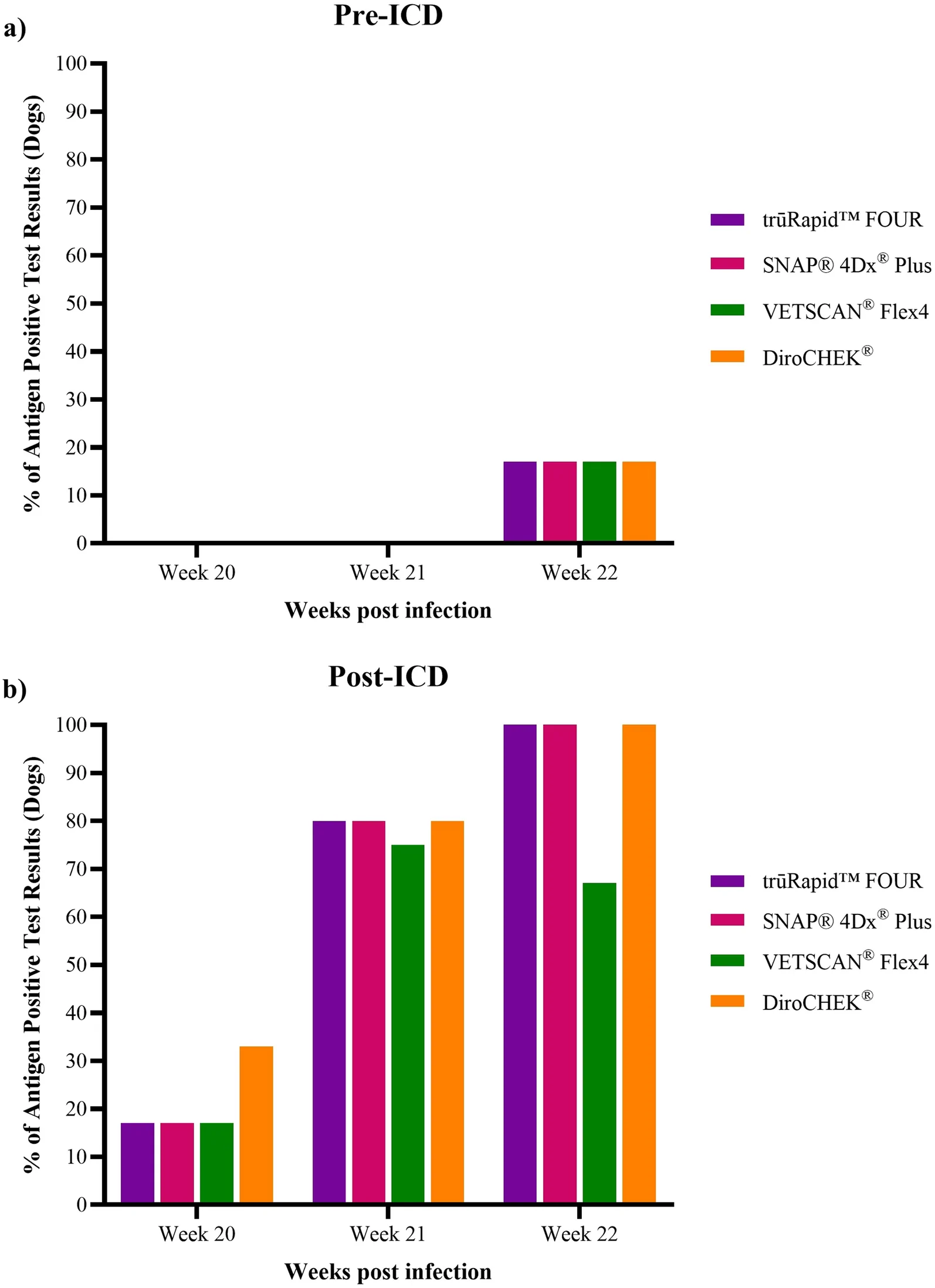
Detection of *Dirofilaria immitis* antigen by four commercial assays. Percent positive antigen results from trūRapid™ FOUR, SNAP^®^ 4Dx^®^ Plus, VETSCAN^®^ Flex4, and DiroCHEK^®^ at weeks 20–22 post-infection, shown **a** pre- and **b** post-immune complex dissociation

No HW antigen was detected by any assay in serum samples collected from weeks 0 to 19, either before or after ICD (Fig. 1). Antigen first became detectable at week 20, but only following ICD. At this time point, trūRapid™ FOUR, SNAP^®^ 4Dx^®^ Plus, and VETSCAN^®^ Flex4 each detected antigen in 1/6 dogs (16.7%) post-ICD, whereas DiroCHEK^®^ detected antigen in 2/6 dogs (33.3%); one dog was uniquely positive on trūRapid™, while the other was positive on SNAP^®^ 4Dx^®^ Plus and VETSCAN^®^ Flex4. Antigen detection increased markedly by week 21 post-ICD. DiroCHEK^®^, trūRapid™ FOUR, and SNAP^®^ 4Dx^®^ Plus each detected antigen in 4/5 available samples (80.0%), while VETSCAN^®^ Flex4 identified 3/4 positive samples (75.0%). Pre-ICD samples remained negative at this time point across all assays. By week 22, antigen became detectable pre-ICD in only one dog (16.7%) across all platforms; however, ICD markedly enhanced detection for all assays. trūRapid™ FOUR, SNAP^®^ 4Dx^®^ Plus, and DiroCHEK^®^ each detected antigen in 6/6 dogs (100%) post-ICD, whereas VETSCAN^®^ Flex4 detected 4/6 (66.7%) post-ICD (Fig. 1). Across all late-stage time points, ICD improved antigen detection sensitivity for all assays tested, with the greatest effect observed near the onset of detectable antigenemia pre-ICD. Overall, trūRapid™ FOUR demonstrated a pattern of antigen detection comparable to SNAP^®^ 4Dx^®^ Plus and DiroCHEK^®^, particularly in ICD-treated samples (Fig. 1).

In Cochran’s *Q* test, a significant difference was observed in the overall differences in positivity across all assays (*P* < 0.001) of dogs that had complete data (*n* = 4/6) (Table 1). With the inclusion of a Bonferroni correction, the post hoc analysis showed no significant differences when comparing each assay using pre-ICD to all other assays with pre-ICD (*P* = 1.000) (Table 2). We also observed no significant differences when comparing each assay in post-ICD to all other assays using post-ICD (Table 2). Finally, we found that when comparing each assay between pre- and post-ICD, there were no significant differences observed across all tests (Table 2).

**Table 1:** Cochran’s *Q* test results comparing four commercially available heartworm diagnostic tests, both pre- and post-immune complex dissociation, individually

Comparison of individual methods	*Q* statistics *df* *P*-value
trūRapid™ pre-ICD	52.753 7 < 0.001*
trūRapid™ post-ICD	
SNAP^®^ 4Dx^®^ Plus pre-ICD	
SNAP^®^ 4Dx^®^ Plus post-ICD	
VETSCAN^®^ Flex4 pre-ICD	
VETSCAN^®^ Flex4 post-ICD	
DiroCHEK^®^ pre-ICD	
DiroCHEK^®^ post-ICD	

* Indicates a significant relationship (*P* < 0.05)

**Table 2:** Post hoc analysis using McNemar’s test for each assay comparison

Post hoc analysis (test 1/test 2)	a	b	c	d	*P*-value
Pre/pre					
trūRapid™ FOUR pre-ICD/SNAP^®^ 4Dx^®^ Plus pre-ICD	1	0	0	11	1.0000
trūRapid™ FOUR pre-ICD/VETSCAN^®^ Flex4 pre-ICD	1	0	0	11	1.0000
trūRapid™ FOUR pre-ICD/DiroCHEK^®^ pre-ICD	1	0	0	11	1.0000
SNAP^®^ 4Dx^®^ Plus pre-ICD/VETSCAN^®^ Flex4 pre-ICD	1	0	0	11	1.0000
SNAP^®^ 4Dx^®^ Plus pre-ICD/DiroCHEK^®^ pre-ICD	1	0	0	11	1.0000
VETSCAN^®^ Flex4 pre-ICD/DiroCHEK pre-ICD	1	0	0	11	1.0000
Post/post					
trūRapid™ FOUR post-ICD/SNAP^®^ 4Dx^®^ Plus post-ICD	8	1	1	2	1.0000
trūRapid™ FOUR post-ICD/VETSCAN^®^ Flex4 post-ICD	7	2	1	2	1.0000
trūRapid™ FOUR post-ICD/DiroCHEK^®^ post-ICD	9	0	1	2	1.0000
SNAP^®^ 4Dx^®^ Plus post-ICD/VETSCAN^®^ Flex4 post-ICD	8	1	0	3	1.0000
SNAP^®^ 4Dx^®^ Plus post-ICD/DiroCHEK^®^ post-ICD	9	0	1	2	1.0000
VETSCAN^®^ Flex4 post-ICD/DiroCHEK^®^ post-ICD	8	0	2	2	0.5000
Pre/post					
trūRapid™ FOUR pre-ICD/truRapid™ FOUR post-ICD	1	0	8	3	0.0078
SNAP^®^ 4Dx^®^ Plus pre-ICD/SNAP^®^ 4Dx^®^ Plus post-ICD	1	0	8	3	0.0078
VETSCAN^®^ Flex4 pre-ICD/VETSCAN^®^ Flex4 post-ICD	1	0	7	4	0.0156
DiroCHEK^®^ pre-ICD/DiroCHEK^®^ post-ICD	1	0	9	2	0.0039

Bonferroni correction (α = 0.003); a = positive in both tests; b = positive in test 1, negative in test 2; c = negative in test 1, positive in test 2; d = negative in both tests

## Discussion

This study evaluated the diagnostic performance of the newly developed trūRapid™ FOUR antigen test for early detection of *D. immitis* infection in experimentally infected dogs and compared its performance to three commercially available platforms: SNAP^®^ 4Dx^®^ Plus, VETSCAN^®^ Flex4, and the DiroCHEK^®^ ELISA. The findings demonstrate that all diagnostic assays performed as expected, detecting antigen in accordance with the onset of antigen production, with detectable responses emerging only after week 20. These results align with the known biology of HW development, in which antigen production typically begins around 5–7 months post-infection once mature female worms are established in the pulmonary vasculature [1, 4, 17].

Across all platforms, heat treatment markedly improved antigen detection, particularly during the first 2 weeks when antigenemia emerged. Following heat treatment at week 20 post-infection, POC assays detected 1/6 dogs as positive, whereas DiroCHEK^®^ detected 2/6; one dog was uniquely positive on trūRapid™, while the other was positive on SNAP^®^ 4Dx^®^ Plus and VETSCAN^®^ Flex4. This discordance suggests variability in assay sensitivity during early antigen detection following ICD, potentially reflecting differences in analytical sensitivity or antigen recovery across platforms. Pre-ICD samples showed minimal reactivity, with only a single dog testing positive across all assays at week 22, indicating consistent cross-platform detection only at later stages of infection without ICD. In contrast, ICD enhanced early diagnostic sensitivity for all platforms by increasing true-positive outcomes that were otherwise masked due to immune complex formation. However, ICD is not recommended for routine screening in dogs according to AHS guidelines due to the need for additional sample processing and specialized equipment, limiting its practicality as well as affecting the accuracy of these POC tests in clinical settings [11]. These findings are consistent with previous research demonstrating that antigen masking can significantly limit the sensitivity of HW antigen assays in early or low-burden infections and that ICD can restore detectability [21, 23–26, 28, 29, 35, 36].

Among the assays evaluated, DiroCHEK^®^ demonstrated the highest sensitivity during the earliest detection point at week 20, followed closely by trūRapid™ FOUR and SNAP^®^ 4Dx^®^ Plus. By week 22, trūRapid™ FOUR performed comparably to DiroCHEK^®^ and SNAP^®^ 4Dx^®^ Plus post-ICD, detecting antigen in all animals tested, indicating that this newly available POC assay is capable of reliably identifying infection once circulating antigen is present at historically detectable levels. These findings are consistent with recent clinical evidence demonstrating that trūRapid™ performs comparably to established POC assays, including SNAP^®^ 4Dx^®^ Plus, in suspected HW cases [37].

While this study offers valuable controlled experimental data, several limitations should be acknowledged. First, because archival samples were used, limited serum volumes restricted the ability to test all assays across every dog and time point; specifically, insufficient sample quantity at week 21 prevented complete POC test evaluation (i.e., serum samples were available from only five dogs at week 21, and of these, only four had sufficient volume for testing with the VETSCAN^®^ Flex4). Second, the relatively small cohort size of complete data (*n* = 4/6), inherent to experimental infection models, limits generalizability and underscores the need for statistical analyses to fully characterize differences in diagnostic performance among assays. This resulted in a significant difference using Cochran's *Q* but was underpowered due to the smaller sample size (*n* = 4/6) to show a difference between the paired tests in the post hoc analysis. Third, the Metairie isolate of *D. immitis* was used in the present study, and any isolate-specific variations that could affect test sensitivity were not evaluated. Additionally, each sample was tested only once, which may not fully account for technical variation. Future studies that include naturally infected dogs with diverse worm burden and isolates, HW preventive histories, and microfilaremia status will be important to establish broader clinical relevance.

## Conclusions

Overall, our results indicate that trūRapid™ FOUR is a reliable diagnostic tool for detecting HW antigen as early as 5 months post-infection once antigenemia is established, and performs comparably to widely used POC assays following ICD. Continued evaluation across diverse clinical settings will help confirm its broader utility.

## Data Availability

Data supporting the main conclusions of this study are included in the manuscript.

## Abbreviations

AHS: American Heartworm Society
ELISA: Enzyme-linked immunosorbent assay
HW: Heartworm
ICD: Immune complex dissociation
OD: Optical density
POC: Point-of-care
